# CircPTK2 inhibits cell cisplatin (CDDP) resistance by targeting miR-942/TRIM16 axis in non-small cell lung cancer (NSCLC)

**DOI:** 10.1080/21655979.2021.2024321

**Published:** 2022-03-01

**Authors:** Yongfu Wang, Yuanlin Wu, Shaoqiang Xie

**Affiliations:** Department of Cardiothoracic Surgery, The Second People’s Hospital of Yibin, Yibin, Sichuna, China

**Keywords:** Circptk2, cisplatin resistance, miR-942, TRIM16, cell progression

## Abstract

In recent years, the problem of cancer resistance has become more and more prominent, seriously affecting treatment efficiency. Circular RNAs (circRNAs) play an important role in cell progression and cancer mechanisms. However, there is a lack of systematic studies on its function in non-small cell lung cancer (NSCLC) resistance. CircPTK2, microRNA-942 (miR-942), and Tripartite motif 16 (TRIM16) levels were detected by Real-time quantitative reverse transcriptase PCR (qRT-PCR). Extracellular acidification rate (ECAR), glucose consumption, and lactate production were assessed using the Seahorse XF96 Glycolysis Analyzer, glucose, and lactate assay kits, respectively. The protein expression was measured with the western bolt Transwell assay was used to determine migration and invasion of transfected cells. (4-5-dimethylthiazol-2-yl)-2,5-diphenyl tetrazolium bromide (MTT) assay and flow cytometry were applied to carry out cell proliferation and apoptosis, respectively. The relationship among circPTK2, miR-942, and TRIM16 were determined by using the dual-luciferase reporter assay and RIP assay. circPTK2 (hsa_circ_0008305) and TRIM16 were low expressed, while miR-942 was significantly highly expressed in NSCLC tissues and cell lines. Moreover, overexpression of circPTK2 remarkably inhibited cell growth, metastasis, and glycolysis in A549/CDDP and H1299/CDDP cells. Promotion of miR-942 or inhibition of TRIM16 could reverse the effects of high circPTK2 expression on cell growth, metastasis, and glycolysis in A549/CDDP and H1299/CDDP cells. CircPTK2 overexpression inhibited the growth of A549/CDDP cells *in vivo*. Furthermore, circPTK2 weakened CDDP resistance of NSCLC through modulating miR-942/TRIM16 axis, providing a novel sight for the treatment of NSCLC and improving the understanding of the CDDP resistance mechanism of NSCLC.

## Graphical Abstract

**Figure:**
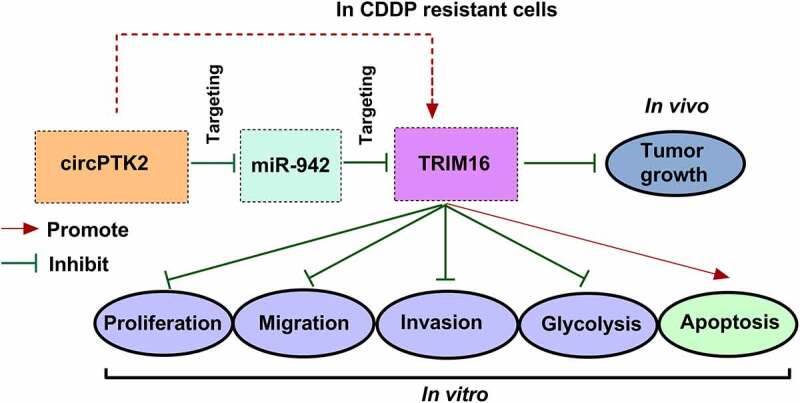

## Background

Lung cancer is one of the malignant tumors with high morbidity and mortality, which poses a great threat to human health and life [1]. Eighty-five percent of them were non-small cell lung cancer (NSCLC) [2]. In the past years, chemotherapy is still the main treatment for NSCLC, and cisplatin (CDDP) is the most common chemotherapy drug for NSCLC treatment [3,4]. Although CCDP-based chemotherapy can improve survival in patients with NSCLC, drug resistance has seriously affected the effectiveness of NSCLC treatment. To improve the survival rate of patients with NSCLC, it is necessary to explore the potential regulatory mechanism of CDDP resistance in NSCLC cells.

CircRNA is a type of non-coding RNA that is abundant in cells and is involved in transcription and translation regulation [5,6]. Studies have shown that the structure of the circRNA molecule is closed-loop and will not be affected by exonucleases, whose expression is more stable in cells [5]. More than that, the function of circRNA has been extensively studied. The most important function is to bind miRNAs as miRNA-sponges and regulate post-transcriptional mRNA expression. The existence of this mechanism has been verified in various diseases, including liver cancer, nonalcoholic steatohepatitis and hepatocellular carcinoma [7–9]. In NSCLC, some regulatory mechanisms of circRNA have been studied, such as circ_0001649, circ_0007534 and circ_0067934, which were related to cell progression [10–12]. In addition, wang et al. reported that hsa_circ_0008305 (circPTK2) was suppressed in NSCLC and regulated TGF-β-induced epithelial-mesenchymal transition and metastasis [13]. However, its function has not been elucidated in the chemo-resistance and treatment of NSCLC.

microRNAs (miRNAs) are also important regulators of cancer development and formation. Many studies have found that miRNAs not only participate in the regulation of cancer development [14,15], but also have important research significance for disease diagnosis and treatment [16–18]. miR-942 has been shown to regulate cell proliferation and apoptosis in esophageal squamous cell carcinoma, human liver fibrosis and NSCLC [19–21]. Consistent with yang et al study [21], we found that miR-942 was highly expressed in NSCLC, meanwhile, miR-942 expressed lowly in NSCLC/CDDP cells compared with that in NSCLC cells, but its related regulatory mechanisms remain unclear.

Tripartite motif 16 (TRIM16) is a member of the TRIM protein family, which plays an important role in cancers. PY Kim et al reported that enhanced TRIM16 expression could promote cell apoptosis in cancer cells, especially neuroblastoma cells [22,23]. Moreover, many studies determined that TRIM16 was an important tumor suppressor in various cancers, including NSCLC, hepatocellular carcinoma, ovarian cancer, and breast cancer, and was related to cell migration, invasion and EMT [24–27]. In this study, we found that TRIM16 expression has changed in NSCLC and NSCLC/CDDP cells, but the specific resistance mechanism of how it is involved in the progression of NSCLC cells is unclear.

In this paper, we predicted the targeting downstream factors of circPTK2 based on bioinformatics and validated the regulatory role and mechanism of circPTK2 in NSCLC based on classic cytological experiments. Here, we aim to verify the role of circPTK2 in the malignant behavior and cisplatin resistance in NSCLC and to clarify whether the involvement of circPTK2 in NSCLC function was mediated by miR-942/TRIM16 axis.

## Materials and Methods

### Patients and tissues

NSCLC tissues and the adjacent non-cancerous tissues were collected from 50 patients who had no adjunctive treatment prior to the surgery in The Second People’s Hospital of Yibin, Yibin between September 2014 and July 2018. And the patient’s informed consent has been obtained. All experiments have been approved by the Ethics Committee of The Second People’s Hospital of Yibin, Yibin. All collected tissues were quickly put into liquid nitrogen before being stored at −80°C.

### Cell cultured and transfection

The NSCLC cell lines (A549 and H1299) and their CDDP-resistant cell lines (A549/CDDP and H1299/CDDP cells), as well as normal lung epithelial cell lines (16HBE), were purchased from the Cell Bank of the Chinese Academy of Sciences. The cell lines were cultured in RPMI 1640 medium (Gibco, Carlsbad, CA, USA) supplemented with 10% fetal bovine serum (FBS) in a humidified atmosphere with 5% CO_2_ at 37°C.

The overexpression of cirPTK2 plasmids (circPTK2) and inhibition TRIM16 plasmids (si-TRIM16#1: 5ʹ-AACCTGCATGGTGAATTACTGTGAA-3ʹ, si-TRIM16#2: 5ʹ-CGCATCAGGTGAACATCAAACTGCA-3ʹ), as well as their negative control (vector or si-con) were constructed and purchased from GenePharma (Shanghai, China). miR-942 inhibitors and mimics (anti-miR-942 and miR-942) were purchased from RiboBio (Guangzhou, China). All plasmids and oligos were transfected into cell lines by using Lipofectamine 3000 (Life-Technologies, Carlsbad, CA, USA) according to the manufacturer’s instructions

### Real-time quantitative reverse transcriptase PCR (qRT-PCR)

Total RNAs from clinical tissues and cell lines were isolated using TRIzol (Thermo Fisher Scientific, Carlsbad, CA, USA) and followed by treatment with 3 U/mg DNase I (Qiagen, Hilden, Germany) at 37°C for 15 min. 2 mg of total RNA was reversed transcribed into cDNA using the PrimeScript® RT reagent Kit (Takara, Shiga, Japan). For the circPTK2 and TRIM16, the Reverse Transcription Reagents (Applied Biosystems, Foster City, CA) were performed to detect their expression. For the miR-942, the TaqMan® MicroRNA Reverse Transcription kit (Applied Biosystems) was used to measure its expression. The relative RNA expressions were calculated by using the 2 ^−ΔΔCt^ method [28]. Expression of β-actin or U6 was used as an endogenous control for TRIM16, circPTK2, or miR-942.

circPTK2 forward:5ʹ-AGAAGGTGAACGGGCTTTG-3ʹ

circPTK2 reverse: 5ʹ-TTTTGGCCTTGACAGAATCC-3ʹ

TRIM16 forward: 5ʹ-CGAGATGGAGAAGAGTAAGCAGG-3ʹ

TRIM16 reverse: 5ʹ-CCCGAGAGTTTATCCTTCAGCC −3ʹ

miR-942 forward: 5ʹ-GCGCGCTCTTCTCTGTTTTGGC −3ʹ

miR-942 reverse: 5ʹ-GTGCAGGGTCCGAGGT-3ʹ

β-actin forward: 5ʹ-GGAAATCGTGCGTGACATTA-3ʹ

β-actin reverse: 5ʹ-GGAGCAATGATCTTGATCTTC −3ʹ

U6 forward: 5ʹ-CGCTTCGGCAGCACATATAC-3ʹ

U6 reverse: 5ʹ-TTCACGAATTTGCGTGTCAT-3ʹ

### Subcellular fractionation assay

Generally, 5 × 10^6^ A549 and H1299 cells were rinsed twice in pre-chilled PBS, followed by suspension in cytoplasm lysis buffer. After being centrifuged for 4 min, the upper solution was transferred into a clean tube and the nuclear pellet was introduced in nucleus lysis buffer. After being isolated by TRIzol (Thermo Fisher Scientific), the isolated RNA was detected using qRT-PCR, normalizing to GAPDH (cytoplasmic control) and β-actin (nuclear control).

### Western blot

The protein level of TRIM16 was detected using Western blot, according to the experimental procedures recorded in the previous publications [29]. Briefly, transfected cells were lysed in Radioimmunoprecipitation assay (RIPA) lysis buffer with protease inhibitor (Thermo Fisher Scientific) to extract total protein. The protein quantification was measured by the BCA^TM^ Protein Assay Kit (Pierce, Appleton, WI, USA). Then the equivalent protein (50 µg) was added to the sodium dodecyl sulfate-polyacrylamide gel electrophoresis (SDS-PAGE) to obtain the target protein. Next, the target proteins in the gel were transferred onto polyvinylidene difluoride (PVDF) membranes and incubated with primary antibodies against TRIM16 (ab72129, 1:2000, Abcam, Cambridge, MA, USA), hexokinase 2 (HK2, ab227198, 1:5000, Abcam), B-cell lymphoma protein 2 (Bcl-2)-associated X (BAX, ab32503, 1:2000, Abcam), Matrix metalloproteinase 2 (MMP2, ab92536, 1:1000, Abcam), Proliferating cell nuclear antigen (PCNA, ab18197, 1:1000, Abcam), and β-actin at 4°C overnight. The membranes were then incubated with second antibodies (Horseradish peroxidase-conjugated IgG antibody). Finally, the Western blot intensities were detected using LAS 4000 Image Reader (Fujifilm, Tokyo, Japan)

### (4-5-dimethylthiazol-2-yl)-2,5-diphenyl tetrazolium bromide (MTT) assay and IC50 detection

Cell proliferation of transfected cells was measured using MTT assay, based on the prior publications [30]. Briefly, transfected cells were seeded into 96 well plates at a density of 5000 cells/well, and added into 20 µL MTT solution (5 mg/ml). After being incubated the plates at 37°C for 4 hours, 150 µL of DMSO was added to each well and incubated at 37°C for 15 min. The absorbance at 490 nm was detected using a microplate reader (BioTek, Winooski, VT, USA) to assess cell proliferation.

The transfected cells were incubated for 24 hours in 96 well plates and then treated with the concentrations of cisplatin (0.01, 0.1, 1, 10, 100, 1000 µM) for 48 h. Next, the MTT solution and DMSO were added into wells respectively. Finally, cell proliferation was detected as the above description of us. IC50 is the concentration at which the cell survival rate is half that of the control sample.

### Cell invasion and migration

This assay was performed as previously described [31]. Cell invasion and migration of transfected cells were detected using transwell assay. For migration assay, 5 × 10^4^ transfected cells in RPMI 1640 medium with FBS were seeded into the upper chamber. For invasion assay, 1 × 10^5^ transfected cells in RPMI 1640 medium with FBS were seeded into the upper chamber with a Matrigel-coated membrane (Corning Life Sciences, Lowell, MA). Then, the lower chamber was added into RPMI 1640 medium with 10%FBS. Incubation for 24 h later, the cells in the upper chamber were removed and strained and counted using a light microscope to assess cell invasion and migration.

### Cell apoptosis

As previously described [32], cell apoptosis of transfected cells was measured using a PI/Annexin V-FITC Apoptosis Detection Kit (BD Biosciences, San Jose, CA). Briefly, transfected cells were double-stained with propidium iodide and fluorescein isothiocyanate (FITC)-Annexin V and then were analyzed using a flow cytometer (Gallios, Beckman, USA) [33]. Also, the caspase 3 activity in A549/CDDP and H1299/CDDP cells was determined using the Caspase 3 Activity Assay Kit (Beyotime, Shanghai, China), based on the user’s guidebook.

### Extracellular acidification assay

In short, 1 × 10^4^ transfected A549/CDDP and H1299/CDDP cells were introduced into the Seahorse XF^e^ 96 Extracellular Flux Analyzer (Seahorse Bioscience, Billerica, MA, USA), followed by baseline measurements. Whereafter, glucose, oligomycin (oxidative phosphorylation inhibitor), and 2-DG (glycolytic inhibitor) were sequentially injected into each well at indicated time points. Finally, ECAR was measured using XF96 Wave software (Seahorse Bioscience).

### Glucose consumption and lactate production assay

For glucose consumption assay, transfected A549/CDDP and H1299/CDDP cells were harvested and the cell supernatant was collected, followed by the assessment of glucose concentration using a glucose assay kit (Sigma-Aldrich, St. Louis, MO, USA), as per the operation manual. Glucose consumption was calculated via subtracting the glucose concentration in the collected medium at the specified time from the glucose concentration in the original fresh medium.

For lactate production assay, the concentration of lactate in the collected cell medium was determined using a lactate assay kit (BioVision, Milpitas, CA, USA). Lactate production was calculated via measuring the lactate concentration in the collected medium at the specified time minus the lactate concentration in the original fresh medium. These assays were performed as previously described [34].

### Dual-luciferase reporter assay

The wild type (WT) circPTK2 or TRIM16 sequences containing the binding sites for miR-942 were inserted into the pGL3 promoter vector (Promega, Madison, WI, USA), named circPTK2-WT or TRIM16-WT, the mutate type (MUT) sequences not containing binding sites for miR-942 were also inserted into pGL3 promoter vector (Promega), named circPTK2-MUT or TRIM16-MUT. Then these vectors were co-transfection with miR-942 or anti-miR-942 and their control (miR-con, anti-miR-con) into A549/CDDP and H1299/CDDP cells by using Lipofectamine 3000 (Life-Technologies) according to the manufacturer’s instructions. After incubation for 48 hours, the luciferase activity of each experiment was measured with the dual-luciferase Reporter Assay System (Promega).

### RNA immunoprecipitation (RIP) assay

The EZ-Magna RIP Kit (Millipore, Billerica, MA, USA) was used in RIP assay [35]. According to the manufacturer’s protocols, cells were lysed in RIP lysis buffer containing proteinase and RNase inhibitors, then were co-incubated with 100 µL RIP lysates and the human anti-Ago2 antibody or mouse IgG antibody. Proteinase K buffer was added to digest the protein. The immunoprecipitated RNAs were extracted and then were used to detect the enrichment of TRIM16 and circPTK2 using qRT-PCR.

### Fluorescence in situ hybridization (FISH)

Briefly, RiboBio (Guangzhou, China) designed and synthesized the oligonucleotide-modified probe sequence for circPTK2 and miR-942. Cell nucleus was counter-stained with 4,6-diamidino-2-phenylindole (DAPI, Cell Signaling Technologies, Danvers, USA) for 30 min. According to the producer’s instructions, the slides were observed by FV10i confocal microscope (Olympus, Tokyo, Japan).

### Animal experiment

The animal experiment was approved by the Ethical Committee for Animal Research of The Second People’s Hospital of Yibin, Yibin. A total of 15 BALB/c female nude mice were purchased from the Hubei Research Center of Laboratory Animal (Wuhan, China). A549/CDDP cells transfected with Mock, LV-con or LV-circPTK2 were subcutaneously injected into mice. Long and short diameters (L and W) of the tumor were measured using an vernier caliper each week. After 5 weeks, the mice were killed and tumor weights were measured. The tumor volumes = length × width ^2^ /2.

### Statistical Analyses

The data analysis was analyzed and performed using GraphPad Prism 7.0 (GraphPad Software Inc., La Jolla, CA). All data were presented as mean ± standard deviation (S.D.). Statistical significance of two groups was analyzed using a Student’s *t*-test, Statistical significance of three or more groups were detected using one-way analysis of variance (ANOVA). *P* values < 0.05 was considered statistical significant.

## Results:

### CircPTK2 was relatively low expressed in NSCLC tissues and cell lines

**Figure 1.**
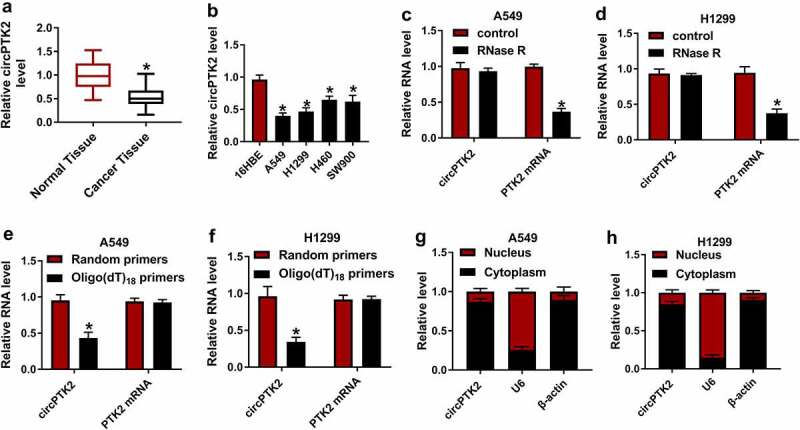
**CircPTK2 (hsa_circ_0008305) was relatively low expressed in NSCLC tissues and cell lines**. (a) Relative expression of circPTK2 in NSCLC tissues and normal tissues. (b) Relative expression of circPTK2 in NSCLC cell lines (A549, H1299, H460 and SW900) and normal cells (16 HBE). (c and d) Relative expression of circRNA and PTK2 mRNA with or without RNase R. (e and f) circPTK2 expression and PTK2 mRNA expression were detected using oligo(dT)_18_ primers than that with Random primers in the reverse transcription products of A549 and H1299 cells. (g and h) The cellular localization of circPTK2 in NSCLC cells was analyzed by Subcellular fractionation assay.*p < 0.05.

To identify the role of circPTK2 in NSCLC, its expression pattern was detected using qRT-PCR assay. As shown in Figure 1(a), the expression of circPTK2 in NSCLC tissue was significantly lower than that in normal tissue. In addition, the expression of circPTK2 in NSCLC cell lines (A549, H1299, H460 and SW900) was significantly lower than that in normal cells (Figure 1(b)) and we selected A549 and H1299 cell lines for the following research. Figure 1(c,d) showed that circPTK2 is stable and insensitive to RNase R. In addition, circPTK2 expression was significantly lower with Oligo(dT)_18_ primers than that with Random primers, but PTK2 mRNA expression with Oligo(dT)_18_ primers have no effect on that with Random primers in A549 and H1299 cells (Figure 1(e,f)). Besides, circPTK2 was predominantly located in the cytoplasm of A549 and H1299 cells, suggesting the potential post-transcriptional regulatory mechanism of circPTK2 in NSCLC cells (Figure 1(g,h)). These results suggested that circPTK2 played roles in NSCLC.

### Overexpression of circPTK2 reduced CDDP resistance in A549 and H1299 cell lines

**Figure 2.**
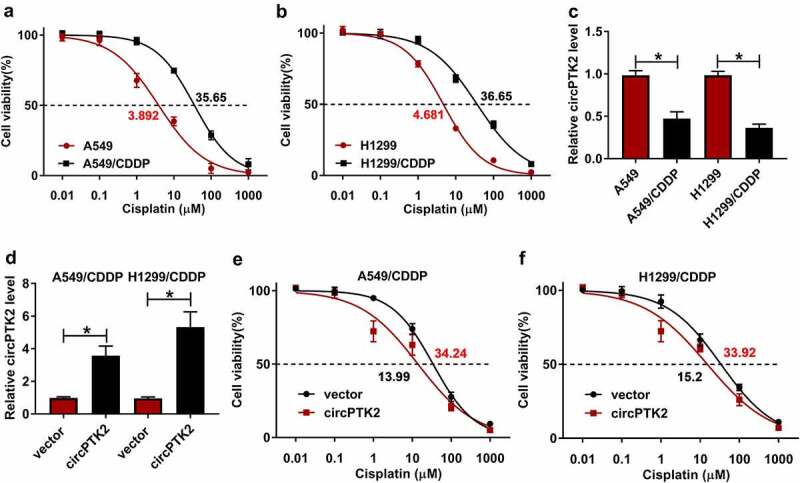
**Overexpression of circPTK2 reduced CDDP resistance in A549 and H1299 cell lines**. (a and b) Determination of IC50 in A549, A549/CDDP, H1299 and H1299/CDDP at the concentrations of cisplatin (0.01, 0.1, 1, 10, 100, 1000 µM). (c) The expression of circPTK2 was detected in A549, A549/CDDP, H1299 and H1299/CDDP. (d) The expression of circPTK2 was detected in vector and circPTK2 groups of A549/CDDP and H1299/CDDP. (e and f) Determination of IC50 in vector and circPTK2 groups of A549/CDDP and H1299/CDDP at the concentrations of cisplatin (0.01, 0.1, 1, 10, 100, 1000 µM). *p < 0.05.

In order to further understand the function of circPTK2 in NSCLC cells, overexpressed circPTK2 cell lines were constructed and stably expressed in A549/CDDP and H1299/CDDP cell lines (Figure 2(d)). In the experiment, we found that the cell viability was decreased with the increase of CDDP concentration (Figure 2(a,b,e,f)). Moreover, the IC50 of A549/CDDP and H1299/CDDP cells were significantly higher than that of A549 and H1299 cells. The expression of circPTK2 was decreased in A549/CDDP and H1299/CDDP cells compared A549 and H1299 cells (Figure 2(c)). Moreover, circPTK2 overexpression effectively reduced IC50 of A549/CDDP and H1299/CDDP cell lines (Figure 2(e,f)), implying that circPTK2 overexpression could decrease CDDP-resistance of NSCLC.

### Overexpression of cirPTK2 inhibited cell growth in A549/CDDP and H1299/CDDP cells

**Figure 3.**
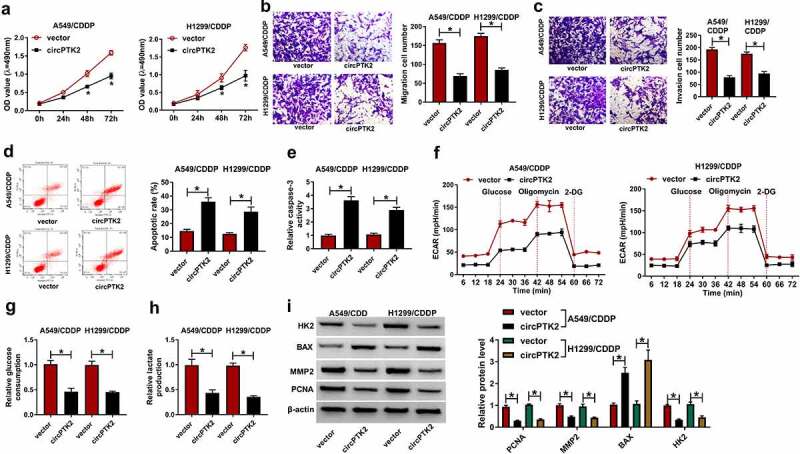
**Overexpression of cirPTK2 inhibited cell growth in A549/CDDP and H1299/CDDP cells**. (a) Cell proliferation was measured in vector and circPTK2 groups of A549/CDDP and H1299/CDDP using MTT assay. (b and c) Cell migration and invasion were measured in vector and circPTK2 groups of A549/CDDP and H1299/CDDP using Transwell assay. (d) Cell apoptosis was measured in vector and circPTK2 groups of A549/CDDP and H1299/CDDP using flow cytometry. (e) Caspase-3 activity analysis in vector or circPTK2-transfected A549/CDDP and H1299/CDDP cells. (f) Extracellular acidification rate (ECAR) analysis presented the glycolytic capacity of A549/CDDP and H1299/CDDP cells transfected with vector and circPTK2. (g and h) Glucose consumption and lactate production were detected in vector and circPTK2 groups of A549/CDDP and H1299/CDDP using glucose and lactate assay kits. (i) HK2, BAX, MMP2, and PCNA protein levels were determined in vector and circPTK2 groups of A549/CDDP and H1299/CDDP using Western blot assay. *p < 0.05.

In order to further study the effect of circPTK2 on the growth of A549/CDDP and H1299/CDDP cells, we measured the cell proliferation, migration, invasion, and apoptosis in each group. MTT results showed that the overexpression of circPTK2 significantly inhibited the proliferation of A549/CDDP and H1299/CDDP cells (Figure 3(a)). The results of transwell analysis showed that migration and invasion of A549/CDDP and H1299/CDDP cells were significantly reduced in the circPTK2 group, compared with the vector group (Figure 3(b,c)). Meanwhile, increasing the expression of circPTK2 significantly increased the apoptosis rate of A549/CDDP and H1299/CDDP cells (Figure 3(d)). Similar to the flow cytometry results, the upregulation of circPTK2 elicited an apparent enhancement in caspase-3 activity in A549/CDDP and H1299/CDDP cells (Figure 3(e)). Additionally, to evaluate the effect of circPTK2 on glycolysis in CDDP-resistant cells, we examined the glycolytic capacity in A549/CDDP and H1299/CDDP cells. As illustrated in figure 3(f), transfection of circPTK2 led to a substantial decline in ECAR in A549/CDDP and H1299/CDDP cells. Meanwhile, we also analyzed the relevant indicators of glycolysis in A549/CDDP and H1299/CDDP cells. Data exhibited that glucose consumption and lactate production were notably reduced after the overexpression of circPTK2 in A549/CDDP and H1299/CDDP cells (Figure 3(g,h)). In addition, Western blot assay also showed that the upregulation of circPTK2 resulted in a significant decrease in HK2 (glycolytic enzyme), MMP2 (migration/invasion marker), PCNA (proliferation marker), and an evident increase in BAX (apoptosis marker) in A549/CDDP and H1299/CDDP cells (Figure 3(i)). All results showed that increased circPTK2 expression significantly suppressed cell growth, metastasis, and glycolysis of A549/CDDP and H1299/CDDP cells in NSCLC.

### miR-942 was negatively regulated by circPTK2 in A549/CDDP and H1299/CDDP cells

**Figure 4.**
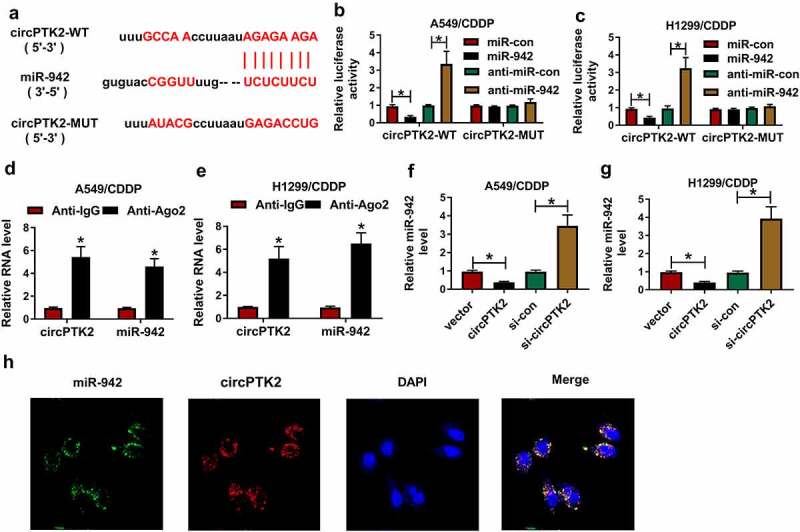
**miR-942 was a target miRNA of circPTK2 in A549/CDDP and H1299/CDDP cells**. (a) Circular RNA Interactome results showed a high binding site sequence between circPTK2 and miR-942. (b and c) Luciferase activity was measured when the miR-942 bound to circPTK2 in A549/CDDP and H1299/CDDP. (d and e) circPTK2 and miR-942 existed in the production precipitated by anti-Ago2 in A549/CDDP and H1299/CDDP. (f and g) the relative expression of miR-942 was detected in A549/CDDP and H1299/CDDP. (h) The cellular localization of circPTK2 and miR-942 in NSCLC cells was analyzed by FISH assay. *p < 0.05.

In order to further verify the chemoresistance mechanism of circPTK2 in cancer cells, we predicted that hsa_circ_0008305 (circPTK2) and miR-942 had targeted binding sites through the Circular RNA Interactome (Figure 4(a)). The validation of the dual-luciferase reporter assay showed that overexpression of miR-942 significantly inhibited the relative luciferase activity of circPTK2-WT, and conversely, low expression of miR-942 significantly increased the relative luciferase activity of circPTK2-WT in A549/CDDP and H1299/CDDP cells. Either miR-942 transfection or anti-miR-942 transfection had no effect on the luciferase activity of circPTK2-MUT group (Figure 4(b,c)). The results of RIP also indicated that circPTK2 could bind to miR-942 in A549/CDDP and H1299/CDDP cells (Figure 4(d,e)). In addition, miR-942 expression was significantly inhibited by circPTK2 transfection but induced by si-circPTK2 in A549/CDDP and H1299/CDDP cells (figure 4(f,g)). What’s more, we found that circPTK2 and miR-942 were mainly distributed in the cytoplasm of NSCLC cells (Figure 4(h)). Therefore, these results indicated that circPTK2 was negatively correlated with miR-942.

### TRIM16 was a target mRNA of miR-942 in A549/CDDP and H1299/CDDP cells

**Figure 5.**
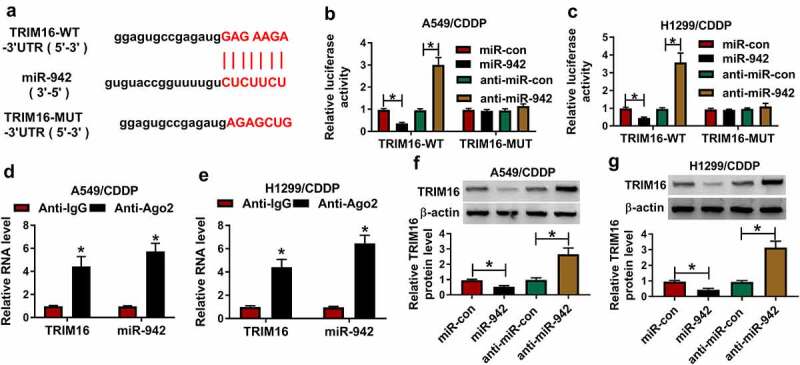
**TRIM16 was a target mRNA of miR-942 in A549/CDDP and H1299/CDDP cells**. (a) microT-CDS results showed a high binding site sequence between TRIM16 and miR-942. (b and c) Luciferase activity was measured when the miR-942 bound to TRIM16 in A549/CDDP and H1299/CDDP. (d and e) TRIM16 and miR-942 existed in the production precipitated by anti-Ago2 in A549/CDDP and H1299/CDDP. (f and g) the relative protein expression of TRIM16 was detected in A549/CDDP and H1299/CDDP. *p < 0.05.

To understand the mechanisms by which miR-942 in NSCLC progression, bioinformatics software microT-CDS was used and results predicted the binding sites between miR-942 and TRIM16 (Figure 5(a)). Also, we found that the expression level of TRIM16 in NSCLC tissue was significantly reduced compared with that in normal tissue (Fig S1A). When miR-942 transfection or anti-miR-942 transfection was combined with TRIM16-WT, luciferase was significantly decreased or increased in A549/CDDP and H1299/CDDP cells (Figure 5(b,c)). RIP assay also verified that miR-942 could bind with TRIM16 in A549/CDDP and H1299/CDDP cells (Figure 5(d,e)). Western blot results showed that protein expression of TRIM16 was significantly inhibited or promoted by overexpression of miR-942 or inhibition of miR-942 in A549/CDDP and H1299/CDDP cells (figure 5(f,g)). Therefore, TRIM16 was a target mRNA of miR-942 in A549/CDDP and H1299/CDDP cells.

### Promotion of miR-942 or inhibition of TRIM16 could reverse the effects of high circPTK2 expression on cell growth in A549/CDDP and H1299/CDDP cells

**Figure 6.**
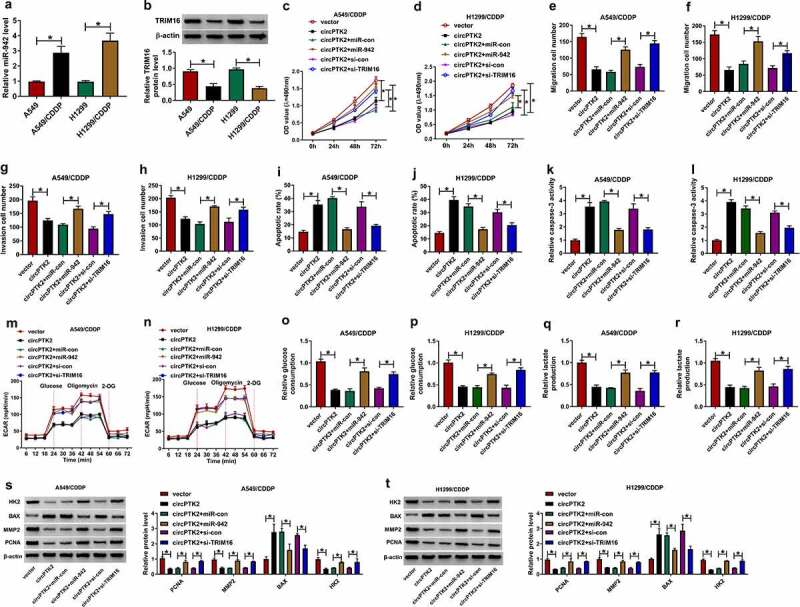
**Promotion of miR-942 or inhibition of TRIM16 could reverse the effects of high circPTK2 expression on cell growth in A549/CDDP and H1299/CDDP cells**. (a and b) The relative mRNA and protein expression of TRIM16 was measured in A549, A549/CDDP, H1299 and H1299/CDDP cell lines. (c and d) Cell proliferation was detected in vector, circPTK2, circPTK2+ miR-con, circPTK2+ miR-942, circPTK2+ si-con, circPTK2+ si-TRIM16 groups in A549/CDDP and H1299/CDDP. (E to H) Cell migration (e and f) and invasion (g and h) were detected in vector, circPTK2, circPTK2+ miR-con, circPTK2+ miR-942, circPTK2+ si-con, circPTK2+ si-TRIM16 groups in A549/CDDP and H1299/CDDP. (i and j) Cell apoptosis was measured in vector, circPTK2, circPTK2+ miR-con, circPTK2+ miR-942, circPTK2+ si-con, circPTK2+ si-TRIM16 groups in A549/CDDP and H1299/CDDP. (k and l) Caspase-3 activity was analyzed in vector, circPTK2, circPTK2+ miR-con, circPTK2+ miR-942, circPTK2+ si-con, circPTK2+ si-TRIM16 groups in A549/CDDP and H1299/CDDP. (m-r) ECAR, glucose consumption, and lactate production were assessed in vector, circPTK2, circPTK2+ miR-con, circPTK2+ miR-942, circPTK2+ si-con, circPTK2+ si-TRIM16 groups in A549/CDDP and H1299/CDDP. (s and t) The protein levels of HK2, BAX, MMP2, and PCNA protein levels were determined in vector, circPTK2, circPTK2+ miR-con, circPTK2+ miR-942, circPTK2+ si-con, circPTK2+ si-TRIM16 groups in A549/CDDP and H1299/CDDP. *p < 0.05.

To further understand the detailed regulatory mechanism of circPTK2. First, we measured the expressions of miR-942 and TRIM16 in A549 and A549/CDDP or H1299 and H1299/CDDP cells. The results showed that the expression of miR-942 was significantly increased in A549/CDDP and H1299/CDDP cells, while the protein expression of TRIM16 was significantly decreased (Figure 6(a,b)). In addition, the transfection efficiency of si-TRIM16#1 and si-TRIM16#2 was detected and shown in Figure S1B. Si-TRIM16#1 showed the higher fold change, so we selected it for subsequent experiments. The MTT assay indicated that overexpression of circPTK2 significantly inhibited the cellular activity of A549/CDDP and H1299/CDDP, but this phenomenon was reversed by overexpression of miR-942 or inhibition of TRIM16 (Figure 6c and 6d). Cell migration and invasion were inhibited by circPTK2 transfection in A549/CDDP and H1299/CDDP cells, which were weakened by higher expression of miR-942 or lower expression of TRIM16 (Figure 6e to 6h). In addition, increasing miR-942 expression or decreasing TRIM16 expression significantly weakened the promoting effect of circPTK2 transfection on A549/CDDP and H1299/CDDP cell apoptosis (Figure 6i and 6j), accompanied by declined caspase-3 activity (Figure 6k and 6l). In terms of glycolysis, the inhibitory of ECAR, glucose consumption, and lactate production caused by circPTK2 overexpression was overturned by miR-942 mimic or si-TRIM16 in A549/CDDP and H1299/CDDP cells (Figure 6m-6r). Synchronously, we also detected proliferation, metastasis, apoptosis, or glycolysis-related markers in A549/CDDP and H1299/CDDP cells. As presented in Figure 6s-6t, miR-942 up-regulation or TRIM16 knockdown could abolish circPTK2-mediated decrease in HK2, MMP2, PCNA protein levels, and increase in BAX protein level in A549/CDDP and H1299/CDDP cells. Therefore, circPTK2 regulated TRIM16 by binding to miR-942 to affect the cell growth, metastasis, and glycolysis of A549/CDDP and H1299/CDDP cells

### CircPTK2 overexpression inhibited the growth of A549/CDDP cells in vivo

**Figure 7.**
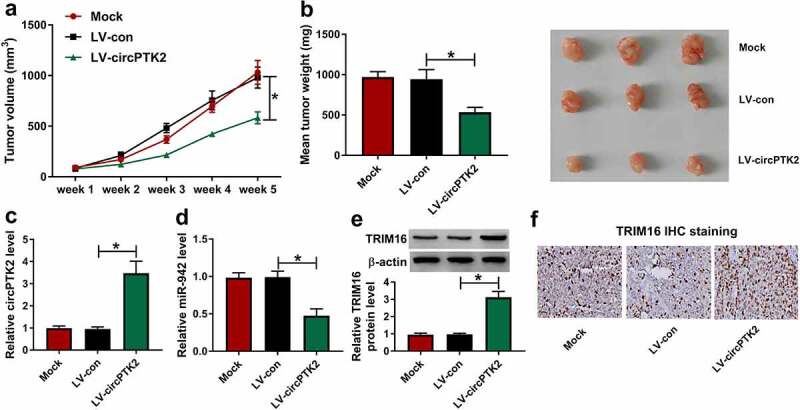
**CircPTK2 overexpression inhibited the growth of A549/CDDP cells in vivo**. (a and b)The tumor volumes and tumor weight of Mock, LV-con and LV-circPTK2 groups were measured in vivo. (C to E) The relative expression of circPTK2 and miR-942, as well as the protein expression of TRIM16 was detected in Mock, LV-con and LV-circPTK2 groups. *p < 0.05.

To further explore the effects of circPTK2 *in vivo*, the nude mice were subcutaneously injected with A549/CDDP cells with or without stable circPTK2 overexpression. The data showed that the tumor volumes of the LV-circPTK2 group were significantly lower than that of LV-NC and Mock groups, especially at week 5 (Figure 7a). Otherwise, the tumor weight of LV-circPTK2 was also notably smaller than that of LV-con and Mock groups (Figure 7b). These results determined that the promotion of circPTK2 could decrease CDDP resistance of A549 cells and inhibit the growth of A549/CDDP cells *in vivo*. Moreover, the expression of circPTK2 and the protein expression of TRIM16 were significantly increased while the expression of miR-942 was inhibited in LV-circPTK2 group, compared with that in LV-con and Mock groups (Figure 7c to 7e). These results indicated that overexpression of circPTK2 could decrease the CDDP resistance of the tumor.

## Discussion

With the development of sequencing technology, a large number of circRNAs have been found to be abnormally expressed in cancers cells, providing new ideas for the treatment and mechanism research of diseases [36–38]. circRNA is a circular RNA that is relatively stable expressed in cells and plays an important role in human diseases, including cancer and neurological diseases [39,40]. Studies have shown that circRNA has important regulatory functions in breast cancer, such as circMTO1 and circANKS1B [41–43]. Accumulating studies reported that circRNA is also involved in cell progression and proliferation in cancers, including NSCLC [44,45]. Otherwise, circRNA is also closely related to CDDP resistance. For example, overexpression of circPVT1 could enhance doxorubicin and cisplatin resistance of osteosarcoma cells by regulating ABCB1 [46]. Although the research of chemo-resistance mechanisms is beneficial to improve the treatment and efficiency of diseases, related research remains scarce. In this study, we found that circPTK2 was involved in the CDDP resistance mechanism of NSCLC. Moreover, circPRK2 had been reported to play a key role in other cancers. For example, circPTK2 was the differential expression in bladder cancer and improved its proliferation and migration [47]. Meanwhile, circPTK2 also was related to metastatic in colorectal cancer and acted as a novel therapeutic target for cancer [48]. Additionally, it has been reported that tumor cells always prefer aerobic glycolysis metabolism (also known as the Warburg effect) to obtain energy. That is, glycolysis can provide sufficient energy for cell proliferation and indirectly reflect the degree of cell metabolism [49,50]. In this paper, we further verified that circPTK2 up-regulation could inhibit cell growth, metastasis, and glycolysis of NSCLC/CDDP cells *in vivo*. Consistently, the suppressive effect of circPTK2 overexpression on NSCLC/CDDP cell growth was also verified *in vitro*. These results implied that upregulated circPTK2 expression decreased the CDDP resistance of NSCLC and enhanced the sensitivity of NSCLC cells to CDDP.

The regulatory network of circRNA has been shown to bind miRNA to regulate mRNA expression in various cancers. For example, circRNA_103808 affected colorectal cancer cell proliferation and migration by binding to the miR-543-3p/FOXO4 axis [51]. In this study, miR-942 has been proven to be a target miRNA for circPTK2 in NSCLC/CDDP cells by a dual-luciferase reporter assay. Some studies have shown that miR-942 mediated cell activation, proliferation, metastasis, and glycolysis in cancers [52–54]. In this paper, miR-942 overexpression significantly reduced the effects of circPTK2 overexpression on A549/CDDP and H1299/CDDP cells. However, its regulatory network also played an important function in cancers. MiR-942 targeted RRM2B to affect cell growth in hepatocellular carcinoma and regulated BARX2 to modulate NSCLC cell progression [21,55].

In this paper, we found that miR-942 directly targeted TIRM16 in NSCLC/CDDP cells, and thought that TIRM16 was a new target of miR-942 in NSCLC. TRIM16 is an E3 ubiquitin ligase that plays an important role in the transcription and apparent modification of mRNA [23]. TRIM16 has been proven to be an important tumor suppressor, which affects the proliferation, autophagy of cancer cells [22,56,57]. Some studies have also shown that TRIM16 can be used as a drug target in melanoma [58,59]. The focus of research on drug resistance has not yet begun. In our study, we verified that inhibited TRIM16 expression could reverse the effects of high circPTK2 expression on cell growth, metastasis, and glycolysis in A549/CDDP and H1299/CDDP cells. Therefore, we determined that circPTK2 weakened the CDDP resistance of NSCLC through modulating the miR-942/TRIM16 axis.

## Conclusion

It is well known that multiple miRNAs co-regulate mRNA or a single miRNA regulates multiple mRNAs in the miRNA/ mRNA regulatory network. The regulation pattern of circRNA/miRNA is similar to that of miRNA/mRNA. In NSCLC, TRIM16 has been verified to be a target gene of miR-135 and is involved in the sensitivity of NSCLC to Gefitinib [60]. This evidence indicated that the circRNA/miRNA/mRNA regulatory network plays a non-negligible role in the drug resistance mechanism of NSCLC.

## Supplementary Material

Supplemental Material

## Data Availability

All data generated or analyzed during this study are included in this article.

## List of abbreviations

NSCLC: non-small cell lung cancer

CDDP: cisplatin

TRIM16: Tripartite motif 16

circPTK2: hsa_circ_0008305

## Highlights

1. CircPTK2 decreased CDDP resistance and suppressed cell growth in CDDP-resistant cells.
2. CircPTK2 was directly bound with miR-942.
3. TRIM16 was a direct target of miR-942.
