# Supplementary figures and images for: CircPTK2 inhibits cell cisplatin (CDDP) resistance by targeting miR-942/TRIM16 axis in non-small cell lung cancer (NSCLC)
Source: Bioengineered. 2022 Mar 1;13(2):3651–64. doi: 10.1080/21655979.2021.2024321 (PMC8973636; doi:10.1080/21655979.2021.2024321)

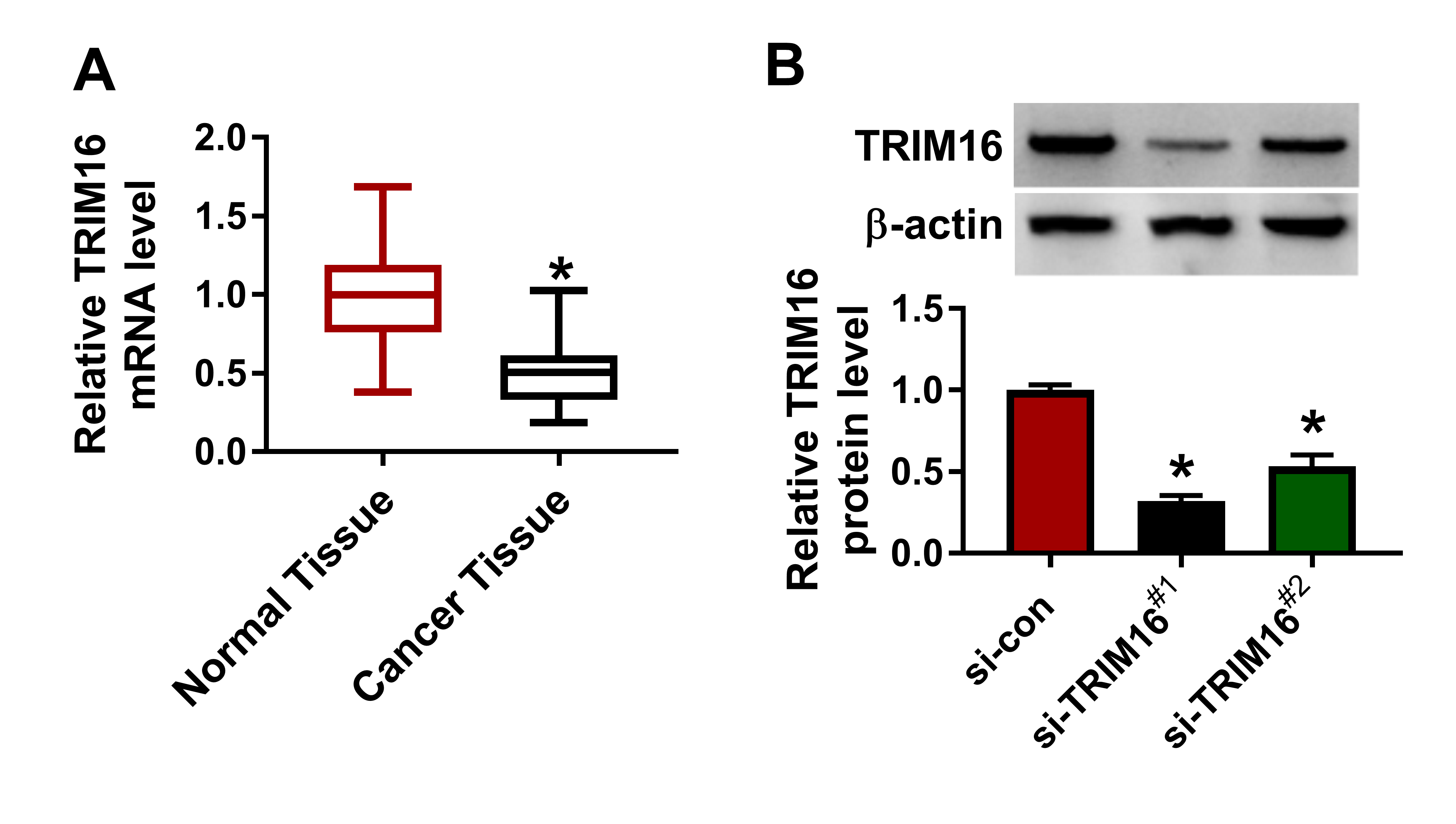

Supplement: Supplemental Material [file KBIE_A_2024321_SM0914.zip › supplementary/Sup Fig 1.tif]
